# Enhancing resilience and self-efficacy in the parents of children with disabilities and complex health needs

**DOI:** 10.1017/S1463423619000112

**Published:** 2019-04-11

**Authors:** M. Whiting, A.S. Nash, S. Kendall, S.A. Roberts

**Affiliations:** 1 WellChild Professor of Community Children’s Nursing Centre for Research and Primary Care, University of Hertfordshire, Hatfield, Hertfordshire, UK; 2 Formerly Senior Research Fellow, Centre for Research and Primary Care, University of Hertfordshire, Hertfordshire, UK; 3 Professor of Community Nursing and Public Health, Centre for Health Services Studies (CHSS), University of Kent, Canterbury, Kent, UK; 4 Formerly Director, Centre for Research in Primary and Community Care, University of Hertfordshire, Hertfordshire, UK; 5 Senior Lecturer, School of Health and Social Care, University of Hertfordshire, Hertfordshire, UK

**Keywords:** children, complex health needs, disability, parenting, resilience, self-efficacy

## Abstract

**Aim:**

The principal aim of this study was to develop, pilot and evaluate an intervention intended to support the development of resilience and self-efficacy in parents of children with disabilities or complex health needs.

**Background:**

Previous research has found that families often experience physical, social and emotional stress in the context of living with and caring for their disabled child. The literature indicates that a key factor in determining how well the parents of these children cope with their situation may be how resilient and self-efficacious they are.

**Methods:**

A total of 16 parents of children with complex needs and disabilities were engaged in a series of guided conversations delivered during six contact visits with nurse co-researchers (community children’s nurses who had received an intensive three-day preparation programme). The conversations, which were supported with additional material that was designed specifically for use in the study, were based around four key themes: emotional coping, practical coping, support networks and ‘you and your child’. The impact of the intervention was evaluated using both qualitative and quantitative measures.

**Findings:**

When interviewed, parents reported increased self-belief and self-confidence and indicated that they felt better supported and stronger as a result of the intervention. This was consistent with the quantitative evaluation which identified significant improvements on scores for active coping and self-blame on the brief COPE inventory scale and for empathy and understanding and self-acceptance on the TOPSE scale. Scores on the self-report distress thermometer demonstrated a significant reduction in self-reported distress scores at the end of the intervention period.

## Introduction

It is estimated that there are around 800 000 children in England who are disabled – 8% of the child population (Family Resources Survey, 2018). This is a population that is growing (Children and Young People’s Outcome Forum, 2012) and includes children with complex health needs. Although there is some evidence that the United Kingdom experience mirrors increasing prevalence rates of childhood disability and complex needs elsewhere in the world, there is a lack of consistency in how disability is both defined and measured at an international level (Cappa *et al.*, 2015). In the UK, there is a strong focus on the provision of community care for this population of children, based upon social policy developed over the course of the past 60 years (see, for instance Whiting, 2010). Community support to families is provided by a wide range of health, education and social care professionals, including community children’s nurses.

Previous research has revealed that families of children with disabilities or complex health needs can experience physical, social and emotional stress in coping with day-to-day living. The need to provide additional care for their child impacts significantly on quality of life for members of the child’s immediate family and has the potential to affect social engagement, employment, income and family finances, uptake and utilisation of health and social care services and the mental and physical health of family members (Mailik Seltzer *et al*., 2001; Contact-a-Family, 2004a, 2004b; Hewitt-Taylor, 2007a; 2007b; Whiting, 2009; 2013).

However, research has also found that some parents cope more effectively in their new role than others and that this may be attributed to these parents being more resilient, that is having better ability to ‘bounce back’ in the face of adversity (eg, Bayat, 2007; Gerstein *et al*., 2009; Peer and Hillman, 2014). With this in mind, this study set out to develop and pilot a resilience-based intervention for use with parent of children with disabilities and complex health needs. This incorporated a particular focus on parenting self-efficacy which has been defined as an individual’s appraisal of his/her competence and expectation of themselves as a parent (Kendall and Bloomfield, 2005).

### Development of the intervention

A novel intervention, Enhance, was developed specifically for use within the study. Drawing upon an extensive review of the field literature (Peer and Hillman, 2014 provide an excellent review of this area), a number of attributes of resilience were identified as having most relevance to the study population: ability to manage emotions, optimism, self-esteem and self-efficacy, coping strategies and social and external support. The parent–child relationship was also considered to be particularly important. These attributes were incorporated into four key themes: practical coping, emotional coping, support networks and ‘you and your child’, each of which the intervention was designed to enhance and, in so doing, promote resilience.

**Figure 1:**
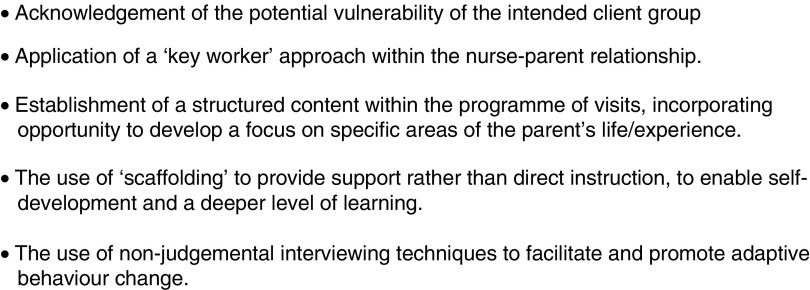
Features of the FNP approach which were considered relevant to this study

In developing the initial proposal for this study, consideration was given to the possible application of learning from the Family Nurse Partnership (FNP), a programme whose central feature is a relationship-based intervention that takes a positive approach to behaviour change (Ball *et al*., 2013). The extensive literature relating to the FNP approach was reviewed, and in addition, opportunity was taken by the Research Team to engage with members of a local FNP service, including visits to accompany staff during their work with families. Several features of the FNP approach were considered to be of relevance to this study (Figure 1) and informed the design of the Enhance intervention which would be delivered through six contact visits, each of approximately 30 min duration over a 12-week period, with the visits to be undertaken by nurse co-researchers (NC-Rs) (see below).

**Figure 2:**
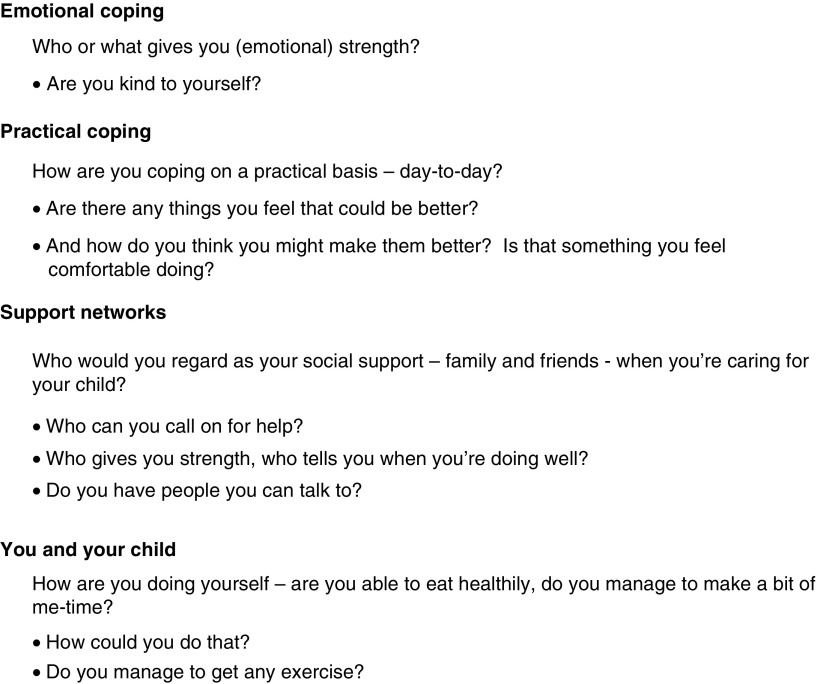
Illustrative questions from each of the four resilience themes

The Enhance intervention consisted a series of guided conversations supported by sets of three to five prompt questions, related to each study theme. Figure 2 provides an illustrative example of the prompt questions for each of the study themes. The conversations were supported with a tool-kit of additional material that was developed specifically for the study. This included practical exercises and resource materials (including contact details for self-help groups and other sources of support and a number of ‘word-games’) which were held in a small folder handed to the parent by the NC-R during the first intervention visit. Further materials were added to the folder as they were introduced during subsequent visits. Members of the study Parent Reference Group (PRG) (see below) played a key role in reviewing the content of the prompt questions, folder and tool-kit as they were developed by the study team.

The Enhance intervention was to be delivered by staff recruited from Community Children’s Nursing Services who received a training programme to prepare themselves for their role as NC-Rs. Service managers at each study sites were appraised of and made a commitment to supporting staff in respect of the workload implications of delivering the intervention. Managers formally endorsed the nomination of NC-Rs to the study. The training programme included a major focus on advanced communication skills and incorporated Good Clinical Practice training to enable the nurses to recruit parents and take informed consent. The NC-Rs were asked specifically to seek to recruit parents to the study whom they considered had the potential to benefit from the intervention.

### Parent Reference Group

In order to ensure the relevance of the proposed study to the families who were the intended focus of the intervention, expert advice was sought from a PRG, which consisted of parents each of whom had prior experience of caring for a child with disabilities or complex health needs. Parents were recruited through the Hertfordshire Parent Carer Involvement Network (http://www.hertsparentcarers.org.uk/) and the WellChild parent network (https://www.wellchild.org.uk/supporting-you/connect-with-others/).

Members of the PRG provided advice and comments by email on aspects of study design, the titles and content of the proposed study themes, and the development of elements of the intervention tool-kit, including the resource sheet of useful websites and contacts. One member of the PRG was a member of the Study Steering Committee.

### Piloting the Enhance intervention

#### Ethical approval

Ethical approval for the study was provided by the East of England – Cambridge and Hertfordshire NRES Committee (Ref: 15/EE/0129) and site-specific approvals were provided for recruitment of both nurses and parents at each study site.

### Nurse co-researchers

A total of four co-researchers (two community children’s nurses, one family support worker and one special school nurse) were recruited from three separate NHS trusts.

## Methods

### Design

The study took a mixed methods approach (Johnson *et al*., 2007), incorporating both quantitative and qualitative data collection and analysis approaches.

### Sample

A total of 16 parents, all mothers, were recruited to the study. They were all the main carers for their children (aged between 10 months and 15 years). Two participants withdrew during the course of the study, but both agreed that data collected up to the point of withdrawal could be included within the data analysis.

### Materials and measures

Quantitative data were collected using four self-completed tools chosen to measure changes in behaviour and attitude and reflecting both outcomes and resilience:

- Distress thermometer (National Comprehensive Cancer Network, 2016)
- Resilience thermometer (developed specifically for this study, modelled upon the distress thermometer and reviewed, during development, by members of the PRG)
- Brief COPE Scale (Carver, 1997)
- Tool to measure parenting self-efficacy (TOPSE) (Kendall and Bloomfield, 2005; Bloomfield and Kendall, 2012).

Semi-structured interview schedules were developed for both parent and NC-R interviews.

### Procedure

Parents provided consent and completed the self-report tools immediately before the first intervention session (T1). The NC-Rs delivered the ENHANCE programme as planned, with occasional delays due to children being admitted to hospital. A mid-point telephone interview was conducted by a member of the research team between the third and fourth intervention visits. A second, face-to-face interview was conducted by a member of the research team approximately 2 weeks after the final intervention visit. All interviews were recorded using a digital audio recorder. Parents completed the self-report tools for a second time (T2) immediately before the second interview. The research team undertook face-to-face interviews with the NC-Rs approximately 2 weeks after they had completed their final intervention visit.

### Data analysis

Interviews were transcribed verbatim. Qualitative data analysis was undertaken using MAXQDA software. Analysis of both parent participant and NC-R interviews took a thematic approach (Boyatzis, 1988), which incorporated the four previously identified themes, with further themes, for example ‘developing relationship with the nurse’, added as they emerged from the data.

Quantitative data were analysed using SPSS v21; *t*-tests were applied to establish significance of the outcomes.

## Findings

The data were anonymised using alpha-numeric identifiers. NC-Rs were identified by the letters A, B, C and D, and parents by letter that corresponds to the NC-R who recruited the parent and a randomly allocated single-digit number (eg, A3, D4 etc.).

### Quantitative data analysis

Of the 16 parents recruited to the study, only 14 parents completed both pre- and post-test questionnaires. With such a small sample size, the inferential statistical analysis presented in the tables should be treated with some caution.

Scores on the distress thermometer (Table 1) decreased between T1 and T2 (*x*=5.00 to *x*=2.43). A within-groups *t*-test indicated that this was significant (*t*_(13)_=2.6, *P*=0.02), indicating that participants considered themselves to be less distressed after receiving the Enhance intervention.

**Table 1:** Distress/resilience thermometers: pre- and post-test results (*n*=14)

	T1		T2			
Scale	*x*	SD	*x*	SD	df	*P*
Distress thermometer	5.00	(2.88)	2.43	(2.82)	13	0.02*
Resilience thermometer	6.00	(2.29)	7.21	(2.28)	13	0.22

Although scores on the resilience thermometer (Table 1) increased between T1 and T2 (*x*=6.00 to *x*=7.21), this change was not significant. Moreover, parental responses to the interview question ‘*Would you say you are more resilient at the end of and as a result of the programme?*’ were not consistent with changes in their individual scores between T1 and T2, indicating that the tool did not provide a reliable measure of resilience.

The scales within the brief COPE inventory (Table 2) indicate how people are coping with their circumstances. A higher score on any of the scales is representative of a particular behaviour or activity occurring more frequently. For some items on the inventory, higher scores indicate a desirable/positive behaviour change (eg, active coping or positive reframing), whereas for other items, higher scores indicate a less desirable/negative behaviour change (eg, denial or substance use). Between T1 and T2, only two scales yielded statistically significant positive change (active coping scale, *t*_(14)_=−2.84, *P*=0.01; and the self-blame scale, *t*_(14)_=2.10, *P*=0.05).

**Table 2:** Brief COPE inventory: pre- and post-test results (*n*=14)

	T1		T2			
Scale	*x*	SD	*x*	SD	df	*P*
Self-distraction	4.27	(1.33)	4.13	(1.92)	14	0.73
Active coping	5.47	(1.46)	6.47	(1.73)	14	0.01**
Denial	2.47	(1.06)	2.53	(0.74)	14	0.82
Substance use	2.00	(0)	2.00	(0)	14	n/c
Use of emotional support	5.60	(1.45)	4.93	(1.91)	14	0.14
Use of instrumental support	4.40	(1.50)	4.93	(1.62)	14	0.18
Behavioural disengagement	2.47	(1.13)	2.13	(0.52)	14	0.17
Venting	3.67	(1.35)	3.13	(1.06)	14	0.18
Positive reframing	4.87	(1.46)	5.47	(1.85)	14	0.11
Planning	4.93	(1.22)	5.47	(2.17)	14	0.21
Acceptance	6.47	(1.60)	5.80	(1.86)	14	0.31
Religion	2.87	(1.60)	3.13	(1.88)	14	0.16
Self-blame	4.07	(1.49)	3.27	(1.62)	14	0.05*

The overall TOPSE score (Table 3) increased by 16 points from T1 to T2 although this was not statistically significant. Scores across all subscales increased between T1 and T2 with a statistically significant difference for two of the subscales: empathy and understanding, *t*_(13)_=−2.32, *P*=0.037, and self-acceptance, *t*_(14)_=−2.09, *P*=0.056.

**Table 3:** TOPSE: pre- and post-test mean scores (*n*=14)

	T1		T2			
Scale	*x*	SD	*x*	SD	df	*P*
Emotion and affection	52.50	(6.43)	55.07	(5.32)	14	0.11
Play and enjoyment	53.07	(6.17)	53.20	(7.92)	14	0.94
Empathy and understanding	42.79	(16.15)	48.00	(10.93)	14	0.04*
Pressures	41.17	(13.21)	41.80	(12.38)	14	0.89
Self-acceptance	50.90	(6.15)	53.60	(6.65)	14	0.06
Learning and knowledge	48.73	(7.78)	50.00	(7.80)	14	0.59
Total TOPSE score	286.30	(29.58)	302.47	(37.28)	14	0.09

### Qualitative data analysis

#### Findings from the parent interviews

The discussion below incorporates data from both mid-point and final interviews.

1. *Previous experience*
  In relation to their experience before their enrolment in the study, parents identified several factors which seemed to present challenges to their resilience, including emotional turmoil, lack of emotional and physical support and lack of sleep:
*“the sleep deprivation for me is just an absolute nightmare because I’m up constantly with [child]” (A1).*
*“ …constant emotional turmoil and lack of sleep and everything, how it can just really chip away at your resilience” (A4).*
*“yeah, I mean the emotional effect and everything else it can have on people is just … it’s horrific …” (D1).*
  The majority of participants identified family and friends as being important in their lives and the lives of their child; however, when talking about their previous experience, a number of parents referred to the additional caring responsibilities in the context of their sense of being alone. Emotional support, through talking to other families in similar situations to their own, through support groups or social media, was seen as being very valuable. Having someone to talk with who potentially understood the circumstance that a family finds themselves in was also recognised as being important.
2. *The study themes*
  Parents were asked to consider each of the four study themes in turn and were also asked to consider all four themes in-the-round.
  1. Practical coping
    The focus on practical coping was an area that some parents found to be very helpful:
*“before we started this programme we weren’t coping at all practically…. And as the programme has gone through we’ve got a lot more help and I feel like I am being a bit more articulate and able to say that I need help and knowing where I can go, I suppose” (C1).*
    One parent described this as ‘*probably the best one, probably the most beneficial*’ (D3) and others offered examples of how they felt that they were now able to take a more practical problem-solving approach. The development of this more practical approach was related in terms of how it had supported parental emotional coping – and vice-versa.
  2. Emotional coping
    For some parents the opportunity to engage in conversation about emotional coping appeared to have cathartic value in itself:
*“So it’s been kind of a relief to actually accept these emotions” (A5).*
*“It helped, well just for me to help point out how I’m feeling at the current time about everything” (A3).*
*“With absolutely everything that I couldn’t talk to anybody about before, I think was a massive help, because it was things I hadn’t even faced myself before” (D1).*
    Parents described how suppressing emotions, particularly in front of their child, was important: *‘I don’t really show that to (*child*) because as I say he is not really aware of it and I want it to stay that way’* (B1). One parent observed that even though she considered herself to be an emotionally strong person anyway, it was still valuable to be provided with an opportunity to explore emotional coping. Parents placed particular value on the relationship that they had established with the NC-R in the specific context of discussing emotional coping: *‘The relationship between me and the nurse is brilliant’* (D1), *‘the nurse made you feel very at ease so you could easily open up’* (C2).
    Parental views on both the practical coping and emotional comping themes were consistent with the positive improvements in relation to active coping and self-blame scales on the brief COPE inventory.
  3. Support networks
    A number of parents observed how having a child with complex needs impacted upon friendships and some family relationships:
*“…but you’re thrown into a whole new world and you suddenly sort of find out which of your friends and family are still, you know, there for you, … and which of your friends and family aren’t and, you know, which can hurt” (A4).*
    From within the intervention tool-kit, the NC-Rs provided a printed list of possible support groups with which parents might make contact. Parents commented positively on engagement with families, including through formal support groups, who were in similar situations to their own. Social media, particularly Facebook® was also identified as a valuable source of support.
  4. You and your child
    Parents valued the opportunity to discuss the relationship between themselves and their child with the NC-Rs, describing in some detail how the conversations had provided an opportunity for positive reflection on what they and their child had achieved together, and the progress the child had made. Some parents were able to contrast this with the sometimes negative view that they had previously taken of things that the child had not been able to achieve and their own personal responsibility for their ‘*failure*’ (D3). This was consistent with the findings from both the brief COPE and TOPSE scales as detailed earlier.
    Parents described how the child’s health needs had impacted upon their own health and wellbeing, with several parents acknowledging that this had become a secondary consideration – their principal responsibility was to care for their child – ‘*so you kind of forget about yourself* […]’ (B4). However, the intervention visits clearly provided parents with an opportunity to think about themselves and their own needs:
*“I do need time for myself and I do need to step back and have some sort of normality” (A5).*
*“So actually having somebody there saying to you ‘are you making time for yourself and are you having some exercise?’” (A4).*
*“Maybe I need to be a bit more aware of me and where I off-load, I suppose and giving time for me” (C1).*
    Parents also provided specific examples of how this aspect of the intervention had prompted them to take some ‘me-time’.
*“She (the NC-R) took some time to ask someone to come over and discuss it with me, and you know, made me believe in myself. So I’m now at college, do my GCSEs… and then I am going to do my Access Course” (A3).*
*“And I have now, I’ve started doing some Zumba classes and getting myself back into shape a little bit which was nice, it kind of made me think twice” (D2).*
*“I probably do more in the day, so I probably, you know, I will meet for a coffee with a friend and so on and it’s quite nice, you know” (D5).*
3. *Were the Enhance programme themes appropriate?*
  When asked about their views on whether the four theme topics were the correct areas upon which to focus within the intervention visits, parental responses were universally positive: ‘*Yeah, absolutely*’ (A2); ‘*Definitely, definitely*’ (A4); ‘*I think they are the right areas, yeah, definitely*’ (C2); ‘*It’s thought provoking for every area*’ (D2). One parent commented that she found it particularly helpful to know in advance the proposed content of the intervention visits:
*“So it’s nice to say ‘Right, today we’re gonna talk about this and then the following week you talk about this’…. it helps you think…” (B4).*

#### Support materials

Parents commented that the folder which had been given to them during the first visit by the NC-R had already been used as an *aide memoire*, had the potential to be used as a resource in the future or as something that could be shared with a partner. The folders included information/resource sheets provided by the Research Team as well as ‘bespoke’ contact lists to be populated by the parents with additional information, including links to government departments, local resources and charities, provided by the NC-R where appropriate.

The tool-kit also included practical and word-based exercises. Several parents referred to the exercises themselves as a positive or enjoyable experience and described how they had been able to use the word-based exercises to help with problem-solving, looking at things from a different point of view or reflecting on situations. One mother commented:

*“It helped, well just for me to help point out how I’m feeling at the current time about everything” (A3).*

#### Views on the ENHANCE programme overall

The intervention visits were highly valued by parents, many of whom specifically highlighted that this was an opportunity for a conversation with somebody who was not a member of the family or a friend. Parents reported that they had found it valuable simply *‘to talk’* and to have somebody *‘to listen’.* Parents commented positively on the particular skills and abilities of the NC-Rs in conducting the conversations. This added to the potential impact of the intervention in that it made the experience enjoyable and was a factor in supporting the ‘scaffolding’ idea behind the Enhance programme. Analysis of the parent interviews confirmed that this aspect of the programme – the relationship with the NC-R – was greatly appreciated. As one participant observed:

*“I think everybody should have the opportunity to have their nurse for half an hour/an hour to talk” (D1).*

Parents welcomed the guidance and support provided by the NC-Rs and emphasised how *‘beneficial it is for people to realise how strong they actually are’* (A3). Parents described how previously they felt that they should take on coping all by themselves and were reluctant to ask for help, so a programme like Enhance where the support was offered was invaluable. They reported that they were becoming emotionally stronger as a result of the programme, were better able to cope both emotionally and practically, were seeing things differently, and were being more accepting of their situation, recognising that there was no blame to attach to their child’s condition.

When asked whether they felt more resilient as a result of the ENHANCE programme, 10 out of 14 responded positively. Each of the four parents who did not consider themselves to be more resilient as a result of the programme indicated that they felt they were already resilient anyway.

In respect of the structure and process of the programme, parents considered the length of the visits to be appropriate, they appreciated the ‘discipline’ of knowing the anticipated duration of the visit in advance – though they also valued the flexibility that was shown by the NC-Rs in allowing the visits to reach a ‘natural’ end point. Parents identified very clearly the need for the programme to be delivered at times that suit each individual family. Most parents indicated that they would have liked to receive the programme at an earlier point in their child’s journey, possibly within a year of diagnosis or initial discharge from hospital, though this by no means was a consistent view from the participants.

#### Findings from NC-R interviews

The NC-R evaluation of the Enhance intervention was generally very positive. The NC-Rs felt very strongly that the 3-day preparatory training programme was essential and they made a number of suggestions for future programme development, including the use of role-play. They reported that in delivering the intervention they were able to use and adapt the questions and the tool-kit in response to the differing needs of individual parents. They provided detailed feedback to the research team on possible revisions to the questions for use in the future.

The NC-Rs felt that the four study themes were appropriate. However, they also observed that *‘it was different for each family’ (NC-RD)*. The NC-Rs reported that they felt that the programme had improved parents’ practical coping abilities and increased their emotional strength.

The NC-Rs reported that the additional time that they were able to spend with families when delivering the intervention was very much valued by the parents, had provided opportunity for relationship building, increased their own job satisfaction and that the knowledge gained from their involvement in the study had the potential to be carried over into their work with other families.

## Discussion and conclusions

This study sets out to develop an intervention that could support parents of children with complex needs by increasing their resilience and parenting self-efficacy. It is the first study that has utilised a guided-conversation-based intervention specifically intended to enhance resilience for parents of children with complex needs. Analysis of the qualitative data strongly indicates that the intervention was well accepted and appropriate to those needs.

Overall, parents and nurses reported positively on their experience of the intervention which was focussed upon four key themes related in the context of the parental role. Quantitative measurement of distress, resilience, coping and parenting self-efficacy reflected the qualitative responses in some areas but not others. For example, there was a significant decrease in reported distress levels following the intervention and an increase in parenting self-efficacy, although this was only significant in two items (self-acceptance and empathy and understanding). The resilience thermometer was not able to reliably assess resilience. The coping measure demonstrated positive change on nine items and a negative change in three items (negative changes on these three items indicated a ‘positive’ outcome).

The sample of parents was very small (only 14 complete data sets) and therefore the possibility of detecting an effect on a before-and-after basis are somewhat reduced particularly in the absence of randomisation and a powered design. Although the distress thermometer, COPE scale and TOPSE have been previously validated, this was the first time the resilience thermometer had been used. Further development of the resilience thermometer will be required to include redesign of the thermometer, revision of explanatory text and testing with a larger group of parents.

The evidence from the qualitative data that parents valued the intervention as it related to their child, their own emotional state and attending to their own needs is an important finding. The statement from one of the parents that *‘everybody should have the opportunity to have their nurse for an hour/half an hour to talk’* (D1) raises the important consideration of whether the Enhance intervention could be a cost-effective approach to supporting parents in this challenging and often stressful part of their lives. Parents reported reduction in distress and small improvements in parenting self-efficacy and coping. When considered alongside the qualitative data, this would suggest that there may be benefits to the wider NHS and society, for example in terms of reductions in GP visits, prescriptions or mental health referrals. Improvement in quality of life may also lead to cost savings.

Future research using a larger sample and a cluster randomised design is indicated. This will also need to incorporate a more detailed analysis of the workforce and health economic implications of delivering an intervention, which it is acknowledged requires a very significant time commitment in its current format. This might also include consideration of the potential for delivery of the intervention by other staff, for instance health visitors. In a future study it will also be important to analyse the relationship between resilience and parenting self-efficacy. In theory, self-efficacy is informed by self-mastery, vicarious experience, verbal persuasion and stress exposure (Bandura, 1982). Although Schwarzer and Warner (2013) identify self-efficacy as being closely related to resilience this may not contribute to how parents ‘bounce back’ from adversity.

In conclusion, this small-scale study of a new intervention to support parents with a child with complex needs has demonstrated that such an intervention is both needed and valued by parents and is acceptable to them. The nurses in the study also confirmed their commitment to the Enhance intervention and the importance of a nurse being able to offer parents this additional support.
